# Post-Mortem RT-PCR Assay for SARS-CoV-2 RNA in COVID-19 Patients’ Corneal Epithelium, Conjunctival and Nasopharyngeal Swabs

**DOI:** 10.3390/jcm10184256

**Published:** 2021-09-20

**Authors:** Francesco Aiello, Marco Ciotti, Gabriele Gallo Afflitto, Maria Cristina Rapanotti, Bartolo Caggiano, Michele Treglia, Sandro Grelli, Sergio Bernardini, Silvestro Mauriello, Carlo Nucci, Luigi Tonino Marsella, Raffaele Mancino

**Affiliations:** 1 Ophthalmology Unit, Department of Experimental Medicine, University of Rome “Tor Vergata”, 00133 Rome, Italy; gabrielegalloafflitto@gmail.com (G.G.A.); nucci@med.uniroma2.it (C.N.); mancino@med.uniroma2.it (R.M.); 2 Virology Unit, University Hospital “Tor Vergata”, 00133 Rome, Italy; marco.ciotti@ptvonline.it; 3 Department of Onco-Haematology, University of Rome “Tor Vergata”, 00133 Rome, Italy; mariacristina.rapanotti@ptvonline.it; 4 Department of Biomedicine and Prevention, University of Rome “Tor Vergata”, 00133 Rome, Italy; bartolocaggiano@gmail.com (B.C.); michele.treglia@ptvonline.it (M.T.); silvestro.mauriello@ptvonline.it (S.M.); tonino.marsella.marsella@ptvonline.it (L.T.M.); 5 Department of Experimental Medicine, University of Rome “Tor Vergata”, 00133 Rome, Italy; sandro.grelli@ptvonline.it (S.G.); sergio.bernardini@ptvonline.it (S.B.)

**Keywords:** coronavirus, SARS-CoV-2, novel coronavirus, coronavirus disease 2019, COVID-19, corneal scraping, conjunctival swab, nasopharyngeal swab

## Abstract

Severe acute respiratory syndrome coronavirus 2 (SARS-CoV-2) disease has been described to possibly be associated with ocular surface disturbances. However, whether the virus could invade ocular tissues still remains elusive. In the present study, we tried to investigate the post-mortem presence of SARS-CoV-2 RNA in corneal epithelium gathered by patients with an ante-mortem confirmed diagnosis of Coronavirus disease-19 (COVID-19). Cadavers with an ante-mortem confirmed diagnosis of moderate to severe COVID-19 were examined. Clinical and demographic features were retrieved from hospital patients’ notes. For each cadaver, corneal scrapings, conjunctival swabs (CS) and nasopharyngeal swabs (NPS) were collected to perform real-time reverse transcriptase polymerase chain reaction ((RT)-PCR) for SARS-CoV-2. Fourteen consecutive cadavers with an ante-mortem confirmed diagnosis of moderate to severe COVID-19 were examined. The last NPS performed ante-mortem confirmed SARS-CoV-2 infection in 12/14 (85.7%) patients. The mean death-to-swab time (DtS) was 3.15 ± 0.5 (2.10–5.1) h. The post-mortem NPS and CS found positive for SARS-CoV-2 RNA were 9/14 (64.3%) and 3/28 (10.7%), respectively. None of the corneal epithelium scrapes tested positive to RT-PCR for SARS-CoV-2 RNA. These data promote the SARS-CoV-2 as not able to contaminate the post-mortem corneal epithelium, while it can persist in different other structures of the ocular surface (i.e., the conjunctiva). It is reasonable to assume that such a contamination can occur ante-mortem too.

## 1. Introduction

On 31 December 2019, the WHO China Country Office was informed of a cluster of pneumonia cases of an unknown aetiology, detected in Wuhan China [1]. Later on, deep sequencing analysis from lower respiratory-tract samples indicated Severe Acute Respiratory Syndrome-Coronavirus-2 (SARS-CoV-2) as the causative agent of the disease, defined coronavirus disease-2019 (COVID-19) [2].

The clue for human-to-human transmission of the virus was first provided by the rapid worldwide spread of the disease [3,4]. Specifically, while respiratory droplets are reported as the main route of transmission, it is now suspected that the virus can even shed and transmit through the gastrointestinal (GI) tract [5,6], the conjunctiva [7] and the placenta [8,9,10].

The marked heterogeneity of the possible transmission route remarks the composite viral tropism for host tissues, which depends on the diverse systemic expression of the angiotensin converting enzyme-2 (ACE-2).

The ACE2 receptor, a zinc-metalloprotease playing key functions in the renin–angiotensin system (RASS), is the host cell entry receptor for the SARS-CoV-2 [10]. It has been shown to be expressed both in the ocular and in the periocular tissues too [11]. Interestingly, as we reported elsewhere, up to 32% of COVID-19 patients might experience conjunctivitis as either the presenting symptom or as a comorbid event, complicating the course of the disease [7,12,13].

Whether SARS-CoV-2 may colonize ocular tissues still remains obscure. The purpose of this study is to evaluate the post-mortem presence of SARS-CoV-2 RNA in corneal epithelium gathered by patients with an ante-mortem confirmed diagnosis of COVID-19. RT-PCR results from corneal specimens will be compared with the ones obtained by conjunctival and nasopharyngeal swabs, collected by the same patients. Findings may pertain to ophthalmology practice, with a specific focus on aerosol-generating procedures and corneal tissue procurement and transplant. As a secondary aim, risk factors for post-mortem viral contamination of human tissues will be assessed too.

## 2. Materials and Methods

Fourteen consecutive cadavers with an ante-mortem confirmed diagnosis of moderate to severe COVID-19 were examined at the Medicine Legal Department of University of Rome “Tor Vergata” between 22 May and 15 June 2020. The ante-mortem diagnosis of COVID-19 was made as per the European Centre for Disease Prevention and Control criteria [14]. The study protocol was approved by the Ethics Committee of the University of Rome “Tor Vergata” (protocol n°: RS77.20). All patients had given their informed consent to the use of personal information at the time of admission to the hospital. The tenets of the Declaration of Helsinki were followed.

All deceased patients were brought from the ward to a morgue immediately after the death. All samples were gathered in the morgue within 6 h from death.

### 2.1. Pre-Mortem Clinical Features

Time from hospital admission to death, patients’ systemic and ocular symptoms, chest computed tomographic scans, results of nasopharyngeal swabs (NPS) and blood tests, as well as therapeutic regimens, were retrieved from hospital patients’ notes.

### 2.2. Post-Mortem Sampling

For each cadaver, corneal scrapings, conjunctival and NPS were collected. The samples were gathered wearing full personal protective equipment. We collected conjunctival swabs (CS) and corneal scrapings from both eyes in each body. Nasopharyngeal swabs were gathered to evaluate post-mortem SARS-CoV-2 positivity. Samples were jointly collected by the same ophthalmologist and legal medicine doctor for all procedures.

To perform NPS and CS, we used synthetic fibre-flocked swabs to insert into a designated sterile tube with 1 mL of liquid Amies (Copan Diagnostic, Murrieta, CA USA).

After mechanically opening the interpalpebral fissure with a sterile eye speculum, the swab was rubbed into the lower conjunctival sac. The corneal scraping was performed only after the CS collection, in order to limit any cross-contamination among different tissues. We employed a surgical blade n° 15 to remove the superficial corneal epithelial layer, sparing the corneal limbus area to avoid any contamination from conjunctival tissue. The excised specimen was collected by the tip of the blade, to be dabbed onto the swab and finally inserted into the sterile vial.

To collect NPS, the swab was first inserted into the mouth cavity to reach the oropharynx. Then, the same swab was introduced into the nostril parallel to the palate in order to reach the nasopharyngeal cavity. Once located, a rotational movement was applied and then left in place for a few seconds in order to absorb the highest amount of secretions possible. Afterwards, the same process was repeated in the contralateral nostril with the same device.

All swabs were then placed into a sterile container to be flash frozen and then stored at −80 °C until the RT-PCR was performed.

### 2.3. RNA Extraction Technique and Real-Time Reverse Transcriptase PCR

Total RNA was isolated from conjunctival swab and corneal epithelium recovered from post-mortem patients using a modified Chomczyńsky and Sacchi’s protocol adapted for poorly cellular samples [15]. RNA integrity was measured for RNAs extracted from the 23 samples using the NanoDrop 2000 (ThermoFisher, Waltham, MA, USA) according to the manufacturer’s instructions. Real-time reverse transcriptase (RT)-PCR for SARS-CoV-2 determination was carried out manually by using Allplex™ 2019n-CoV assay designed for the qualitative detection of the novel coronavirus in respiratory samples (Seegene, Seoul, South Korea). In the case of nasopharyngeal swabs, RNA extraction and PCR set-up were carried out on NIMBUS (Seegene), an automated liquid handling workstation.

Regarding NPS, RT-PCR was performed on a CFX96TMDx platform (Bio-Rad Laboratories, Inc., Irvine, CA, USA) followed by interpretation by Seegene’s Viewer Software. The Allplex™ 2019n-CoV assay is a multiplex real-time PCR targeting the common envelope (E) gene, and the specific nucleocapsid (N) and RNA-dependent-RNA-polymerase (RdRp) genes, complying with the international validated protocols [16].

Positive and negative controls were included in both cases.

### 2.4. Statistical Analysis

The statistical analysis was performed using SPSS version 26.0 (SPSS Inc., Chicago, IL, USA) Means for continuous variables were compared using the *t* test when the data were normally distributed; otherwise, the Mann–Whitney test was used. Proportions for categorical variables were compared using the *χ*^2^ and Fisher’s exact test as appropriate. For unadjusted comparisons, a 2-sided α of less than.05 was considered statistically significant. All continuous variables were expressed as mean ± SD (range).

## 3. Results

Fourteen cadavers from the Medicine Legal Department, University of Rome “Tor Vergata” were examined. Among them, 8 out of 14 (57.1%) were male. Mean age at death was 76.9 ± 12.3 (range: 47–91) years. All the included cases had received the diagnosis of COVID-19 at admission, demonstrated by NPS positive for SARS-CoV-2 (100.0%) and a CT-scan suggestive of interstitial pneumonia (100.0%). The mean duration from admission to death was 28.4 ± 16.8 (1–59) days.

Clinical, laboratory and radiographic features at patients’ admission and death are summarized in Table 1 and Table 2.

All the values are expressed as raw numbers and percentages. Abbreviations—GI: gastrointestinal; NPS: Nasopharyngeal swab.

High blood pressure (85.7%), diabetes (71.4%), chronic obstructive pulmonary disease (57.1%) and chronic kidney disease (42.9%) were the most common comorbidities in our cohort. Immunosuppressive treatment or chemotherapy had been administered to 7 out of 14 patients (50.0%) prior to the admission due to solid tumours (4/14, 28.6%), haematological malignancies (2/14, 14.3%) or previous organ transplants (1/14, 7.1%).

The patients’ therapeutic treatment regimen included the use of antibiotics and antimycotics in 14/14 (100.0%) and 4/14 (28.6%) cases respectively, generally due to opportunistic superinfection which occurred during hospitalization. Antivirals were prescribed in 12/14 (85.7%) patients (Valaciclovir 1 g/day, or lopinavir/ritonavir 400/100 mg/day, or darunavir/ritonavir 800/100 mg/day). Clinical records showed the use of low molecular weight heparin (Enoxaparin 0.5 mg/Kg subcutaneously once a day) in 14/14 (100.0%) cases, Dexamethasone 6 mg/day and Hydroxychloroquine 400 mg/day in 11/14 (78.6%) and in 6/14 (42.9%) patients, respectively. All the patients (14/14, 100.0%) underwent either non-invasive or invasive ventilation.

The last NPS-performed ante-mortem confirmed SARS-CoV-2 infection in 12/14 (85.7%) patients. The 2 patients who attained a negative result in the last ante-mortem NPS were found to have post-mortem NPS, as well as bilateral CS and corneal epithelium non-contaminated by SARS-CoV-2 viral particles.

At death, 6/14 (42.9%) of the recruited cadavers were hospitalized in the intensive care unit department, 7/14 (50.0%) in the internal medicine/infectious disease department and 1/14 (7.1%) in the emergency department. The main cause of death was reported to be cardiac heart failure in 6/14 victims (42.9%), while pneumonia and septic shock occurred in 3/14 (21.4%) and 5/14 (35.7%) cases, respectively. The mean death-to-swab time (DtS) was 3.15 ± 0.5 (2.10–5.1) h.

The post-mortem NPS (Table 3) and CS found positive for SARS-CoV-2 RNA were 9/14 (64.3%) and 3/28 (10.7%), respectively. Specifically, the positive CS patients were gathered by 2 different bodies. In one of them (patient A), viral RNA was retrieved from swabs performed on both eyes; in the other cadaver (patient B), only the sample from the left eye resulted in being positive for SARS-CoV-2 RNA. In addition, while in patient A gene E (Ct 31.82), gene N (Ct 31.37) and gene RpRd (Ct 31.77) resulted positive, in patient B only gene E (Ct 38.20) was detected. Both patients had positive NPS. None of the 28 corneal epithelium scrapes was shown to be positive to RT-PCR for SARS-CoV-2 RNA.

We then investigated the association between patients’ clinical features at admission and at death, as well as medical treatment, with the risk of positive corneal scraping, nasopharyngeal or conjunctival swab, using both univariate and multivariate logistic regression. However, no statistically significant correlation resulted from the analysis, probably due to the small sample size.

## 4. Discussion

The evidence of ocular involvement in COVID-19 was first reported by Guan’s et al. in a retrospective analysis of cases throughout mainland China. At that time, the “conjunctival congestion” prevalence was rated to be as low as 0.8% [17,18,19].

From then on, more in-depth analysis proved SARS-CoV-2 contamination of tear samples to range from 0% to 32%, regardless of the presence of frank conjunctivitis [7], as well as the virus being able to both induce a cytopathic effect in Vero E6 cells [20] and to extensively infect cultured human conjunctival specimens [21,22,23].

Furthermore, keratoconjunctivitis has been described by both Cheema et al. and Guo et al. as the initial clinical presentation of COVID-19, hence suggesting that not only the conjunctiva, but also the cornea, might be targeted and invaded by SARS-CoV-2 [13,22].

The SARS-CoV-2 tropism for ocular and periocular tissues is justified by the expression on these sites of at least 2 proteins: ACE2 and TMPRSS2, the host cell receptor and the co-receptor, which are crucial in the viral cell entry mechanism [23]. Notably, this evidence resulted from the finding of ACE2 and TMPRSS2 expression profiles, both at the gene (RT-PCR) and at the protein level (Western blot and immunohistochemistry), in animal and human corneal and conjunctival tissue samples [12,24,25,26,27].

In our cohort, 3/28 (10.7%) post-mortem CSs resulted in being positive to SARS-CoV-2 RNA, further corroborating the hypothesis of an ocular involvement during the course of COVID-19. Two studies conducted in vivo are in line with this observation. The first one is a report from Iran, in which 10% of tear samples from patients with severe, laboratory-confirmed COVID-19 were found to be positive to the virus [28]. The second is a cross-sectional study by Arora et al. conducted on patients with moderate to severe COVID-19, where the prevalence of positive CS was as high as 24% [19]. The similarities among the aforementioned results may be easily explained in view of the selection criteria used in patient recruiting. In fact, although our data come from samples gathered from deceased patients, their ante-mortem conditions appeared critical, as was further demonstrated by the provided blood tests analysis (Table 2). Similarly, both in Karimi’s et al. and in Arora’s et al. cohort, only patients with moderate or severe COVID-19 were selected. On the contrary, Azzolini et al. recently proved the presence of SARS-CoV-2 (by means of RT-PCR assay) on 52/91 (57.1%) COVID-19 hospitalized patients’ ocular surface [29]. As directly specified by the authors, the reported high rates of positivity might be explained, among other reasons, by the minimization of the time between sample incubation and processing, which in turn would have induced the viral nucleic acid degradation [29].

Additionally, in patient B, only gene E was detected. While gene E is jointly shared by all the members of the Sarbecoviridae lineage, the presence of an ante-mortem positive NPS strongly suggests that the RT-PCR result on CS is reliable.

None of the corneal scrapings were found to be positive to SARS-CoV-2 RNA. Similarly, in a recently published paper, Bayyoud et al. found no viral contamination of post-mortem COVID-19 patients’ corneal tissue samples [30]. The uncommon corneal involvement during the course of COVID-19 might be justified by some of the properties of healthy human corneas. Among them, the lack of a vascular plexus preventing the hematogenous viral dissemination into the organ, and the presence of antiviral agents into the lacrimal film appear to be the most critical [31,32]. However, SARS-CoV-2 colonization of corneal tissue has been recently demonstrated [33]. In fact, 25% (5/20) of posterior corneal tissues from COVID-19-deceased donors have been shown to coincidently result in a positive result in both a SARS-CoV-2 RT-PCR test and in an immunohistochemical assay [33].

In the same paper, Sawant et al. also observed the presence of SARS-CoV-2 RNA in vitreous samples [33]. This evidence, with the clinical one from Invernizzi et al. [34], further corroborates our seminal speculation on the eventual involvement of posterior chamber structures during the course of COVID-19 [7].

The proportion of post-mortem NPS, found to be positive for SARS-CoV-2 in our cohort, resulted in being as high as 64.3%. This percentage seems to be slightly lower than that observed by Skok et al., but higher than the one reported by Fuest et al [35,36]. In fact, the first group found positive NPS in 22/28 (78.6%) cadavers. The shorter DtS (<2 h) compared to the one of our study may explain the aforementioned difference. On the other hand, Fuest et al. mentioned that only 17.4% post-mortem NPS, and none of the CSs, were positive for SARS-CoV-2 [36]. It must be noted that in the latter series, the mean DtS time was greater than the one we evaluated (18.9 h vs. 3.15 h). In addition, the proportion of positive pre-mortem NPS in the Fuest’s cohort was considerably limited as compared to the one of our cohort (47.8% vs. 85.7%) [36].

All patients included in this study had received a diagnosis of COVID-19 at admission, as documented by the presence of NPS positive for SARS-CoV-2 RNA and CT-scan findings suggestive of interstitial pneumonia. However, we find the distribution pattern of signs and symptoms in our cohort to be different to the ones reported in different papers [1,17]. This finding may be due to both the retrospective nature of the clinical data retrieval and to the small sample size, which appear to be two of the most consistent limitations of the present study.

Among other reasons, it must be considered that the tear film dynamic in the cadaver appears to be more consistently modified than in vivo, due to the absent blinking, the reduced tear supply and the possibly increased tendency towards evaporation. As a result, the eventual tissue contamination by viral particles may not be properly counterbalanced by the local fluid dynamics. In addition, we did not perform any evaluation of viral particle viability. Hence, the presence of a positivity to RT-PCR may imply a generic contamination of tissue, rather than the effect of local viral invasion and shedding. However, the tissue sampling was performed shortly after death. Hence, it might be speculated that all the obtained data represents a reliable picture of ante-mortem viral tissue contamination.

Thus, some clinically relevant conclusions may be derived from the aforementioned data. In fact, ophthalmic aerosol-generating procedures (i.e., air puff tonometry, phacoemulsification [37], pars plana vitrectomy [38]) may be at high risk of viral transmission [39]. On the other hand, despite the aforementioned limitations of the study, our results seem to corroborate Bayyoud et al.’s conclusions [30], where they speculated that corneal tissue procurement and processing for corneal transplantation surgery may be a relatively safe procedure due to the absence of viral corneal contamination [30].

To sum up, our findings strongly promote the importance of post-mortem careful safety procedures, since viral RNA may be still detectable and a potential virulence cannot be ruled out. Hence, future researches are advocated to try to elucidate whether viable viral particles are still present in post-mortem ocular tissues, as such evidence could shed some light on the pathogenic capability of SARS-CoV-2, as well as providing new insights into both the clinical management of the affected patients and into the safety procedures to follow up in ante- and post-mortem settings [40].

## Figures and Tables

**Table 1:** Baseline clinical features of the selected cohort.

Clinical Features	Number of Patients (%)	
**Smoking habits**	4 (28.6%)	
**Clinical Symptoms**		
Fatigue	11 (78.6%)	
Cough	8 (57.1%)	
Coryza	2 (14.3%)	
Anosmia	1 (7.1%)	
Ageusia	2 (14.3%)	
GI disturbances	4 (28.6%)	
Conjunctivitis	0 (0.0%)	
**Clinical Signs**		
Fever	10 (71.4%)	
Positive NPS at admission	14 (100.0%)	
Positive CT scan	14 (100.0%)	
Positive NPS before death	12 (85.7%)	

**Table 2:** Blood test results at baseline and before death.

	Admission	Before Death	*p*
Hemoglobin (g/dL)	10.9 (2.2)	8.7 (0.9)	*
WBC (·10^3^/μL)	8.9 (6.1)	11.3 (6.5)	*
Lymphocytes (·10^3^/μL)	0.9 (0.7)	0.4 (0.3)	-
Neutrophils (·10^3^/μL)	7.7 (5.9)	10.6 (6.3)	*
Monocytes (·10^3^/μL)	0.4 (0.4)	0.3 (0.3)	-
Platelet (·10^3^/μL)	211.7 (63.7)	102.8 (69.9)	*
CRP (mg/L)	93.2 (21.6)	189.9 (36.6)	*
PCT	0.6 (0.5)	5.3 (3.6)	-
Ferritin	1322.1 (188.8)	2736.2 (280.2)	-
LDH (IU/L)	313.0 (86.2)	408.3 (211.6)	-
GOT (IU/L)	49.8 (67.1)	136.5 (14.3)	*
GPT (IU/L)	31.6 (60.2)	88.5 (53.3)	-
BUN (mg/dL)	103.7 (77.6)	95.0 (47.0)	-
Creatinine (mg/dL)	2.1 (1.7)	1.7 (1.2)	-
CK (IU/L)	75.5 (42.9)	112.8 (21.4)	-
CK-MB (ng/mL)	1.7 (1.7)	8.44 (12.9)	-
TnI (ng/L)	92.3 (147.5)	519.1 (134.0)	*
D-dimer (ng/mL)	1908.9 (1895.5)	3653.8 (1739.0)	*
Fibrinogen (mg/dL)	782.9 (422.3)	535.2 (367.3)	-
PT (sec)	13.7 (3.6)	15.5 (2.9)	-
aPTT (sec)	27.4 (12.4)	38.5 (7.5)	*
AT-III (%)	91.3 (28.1)	79.1 (24.0)	-

All the values are expressed as means (standard deviation). Abbreviations. WBC: White blood cell count; CRP: C-reactive protein; PCT: Procalcitonin; LDH: Lactic dehydrogenase; GOT: Glutamic-Oxaloacetic Transaminase; GPT: Glutamic/Glutamate Pyruvic Transaminase; BUN: Blood Urea Nitrogen; CK: Creatine phosphokinase; TnI: Troponin-I; PT: Prothrombin Time; aPTT: activated partial thromboplastin time; AT-III: Antithrombin-III. *: statistically significant; -: not statistically significant.

**Table 3:** Real-time, reverse trancriptase polymerase chain reaction results for severe acute respiratory virus-2 RNA on *post-mortem* nasopharyngeal swabs.

Nasopharyngeal Swab	Gene E Ct	Gene RpRd Ct	Gene N Ct
Patient 1	-	-	-
Patient 2	-	-	-
Patient 3	-	-	-
Patient 4	-	34.23	31.67
Patient 5	-	-	-
Patient 6	-	-	-
Patient 7	-	-	-
Patient 8	-	-	-
Patient 9	13.95	15.28	17.37
Patient 10	-	-	-
Patient 11	-	-	-
Patient 12	28.76	30.53	29.67
Patient 13	20.05	22.31	22.83
Patient 14	19.89	20.99	21.50

Abbreviations: E: Envelope; RdRp: RNA-polymerase-RNA-dependent; N: Nucleoprotein; Ct: threshold cycle.

## Data Availability

The data presented in this study are available on request from the corresponding author.
